# Study on the mechanism and response law of fracture movement on the super-high position hard-thick strata

**DOI:** 10.1038/s41598-023-49584-2

**Published:** 2023-12-22

**Authors:** Guangchao Zhang, Guanglei Zhou, Lei Wang, You Li, Yingshi Gu, Zhi Qu, Xipo Zhao, Maosheng Yin, Fangfang Wang, Lingzhuo Zhang

**Affiliations:** 1 State Key Laboratory of Mining Response and Disaster Prevention and Control in Deep Coal Mines, Huainan, 232001 China; 2 College of Energy and Mining Engineering, Shandong University of Science and Technology, Qingdao, 266590 China; 3 Yankuang Energy Group Company Limited, Yanzhou, 272102 China

**Keywords:** Environmental sciences, Natural hazards, Energy science and technology, Engineering

## Abstract

In this paper, a thick plate structural mechanical model was established for the hard-thick rock strata in the Ordos region, which was characterized by the occurrence of high-energy strong earthquakes caused by the fracture of hard-thick rock strata. Subsequently, based on Vlasov's theory, the evolution process of hard-thick rock strata was analyzed. And the paper validated the analysis results using high-energy mine earthquake and surface subsidence data. The following conclusions were drawn: (1) The hard-thick strata in the cretaceous system will not be broken during the advancing and mining process of the test panel of the Shilawusu coal mine. (2) When the test panel is mined to a distance of two panel widths, no fracture occurred in the lower part of the hard-thick strata, because no separated space was formed. (3) When the test panel was advanced to about 856 m, the hard-thick strata have fractured in a vertical direction. (4) No high-energy mine earthquake event has occurred during mining at test panel, and the amount of surface subsidence is approximately 200 mm. (5) In the mining at test panel, two high-energy mining earthquakes occurred at 837 m, 1153 m away from the initial position of the panel, respectively, and the maximum amount of surface subsidence increased to 1397 mm, which accords with the results of the first and periodic breaks calculated by theory. The research results of this paper are of guiding significance for the study of the breaking law of hard-thick strata under similar engineering geological conditions and disaster pre-control.

## Introduction

The coal strata in China have variable conditions, Due to its formation in different geological historical periods, it is inevitable that high strength, large thickness, and well-integrated stratum will appear in the strata. This type of stratum is widely distributed in Inner Mongolia, Shaanxi, Shandong, and other places in China, and is a common geological structure in the coal mining process. However, as the mining space increases, the overhanging roof area of the hard-thick strata keeps increasing and begins to accumulate a large amount of elastic energy, and when the breaking limit is reached, the hard-thick strata will break and release the accumulated energy^1–3^, it is highly prone to triggering strong mining earthquakes, mine water inrush, roof fall and other strong dynamic disasters^4–8^, which not only increases the probability of underground rock burst and affects coal mine safety production, but also causes strong ground vibrations, posing a threat to buildings, and causing panic among residents near the mining area. In the mining areas of western China, there have been high-energy mining earthquake events occurring in multiple mining faces. Taking the Ordos region of Inner Mongolia as an example, there are thick and hard rock strata mainly medium and fine-grained sandstone in the Cretaceous strata, which have high strength, large thickness and distance from coal seams. In coal mining practice, the hard-thick strata, due to the large breaking distance and wide damage impact, can cause strong mine pressure manifestation including sharp surface subsidence and large energy mine earthquakes, which has caused local government departments to pay high attention and order to stop production and seriously affected the safe and efficient coal mining. Therefore, an in-depth study of the breakage movement law of hard-thick strata in the Cretaceous strata is of great significance to realize the prevention and control of dynamic disasters in hard-thick strata and safe and efficient coal mining.

In recent years, scholars at home and abroad have conducted a lot of studies on the law of breaking movement of thick and hard rock strata, and many useful results have been obtained. Yang et al^9^. studied the displacement and stress distribution of thick and hard key strata based on the medium-thick plate theory, revealed the stress and breakage characteristics of key rock strata, and proposed the basis for the breakage mode of key rock strata. Yu et al^10,11^. studied the destabilization conditions, modes, factors and destabilization mechanisms of thick hard-top rock strata by combining theoretical analysis and field measurements, and proposed a destabilization control method for thick hard-top rock strata. Bu et al^12^. established a mechanical model for the thick and hard roof, analyzing the characteristics of rock fracture, instability, and rock pressure manifestation of the thick and hard roof from the perspective of energy conversion, and verifying it through support pressure; Guo Ying et al^13^. through numerical simulations, the laws and influencing factors of stress, destrata, fracture and energy caused by mining-induced tremors (red-bed breaking) were revealed, and the results were verified by field observations; Wang et al^14^. though fracture and movement of thick and hard strata in high position is the main reason to induce strong mining earthquake and rock burst, characteristic and difference of microseismic in multicoal seam mining under thick and hard rock in high position are analyzed systematically. The above studies provide a useful reference for the study of the fracture mechanics law of thick and hard rock strata. However, due to the significant differences in the physical and mechanical properties of the hard-thick strata and their assigned stratigraphic positions in the strata, coupled with the differences in the boundary support conditions caused by mining, all of them will have significant effects on the law of fracture movement of hard-thick strata. The previous studies mainly focus on the research and analysis of the fracture law of the thick and hard rock strata itself, but there is less research on the fracture mechanism and the corresponding initial and periodic fracture response law of the thick and hard key Cretaceous strata in the Ordos area under the conditions of large space mining with multiple working faces.

Many scholars have conducted extensive research on the mechanisms of multi-face and large space mining, as well as the instability and damage caused by thick and hard rock formations. At present, some scholars have used theoretical analysis, numerical simulation, on-site measurement, and other methods to study the occurrence mechanisms of thick and hard rock stratum fractures, high-energy mining earthquakes, and surface subsidence, and have achieved fruitful results. Bai and Cao et al^15–17^. established a structural mechanics model for hard-thick strata slabs and studied and analyzed the correlation between thickness variation, instability forms, energy variation, and mine seismicity in hard-thick strata. Zhang et al ^18–20^. studied the vibration damage to surface buildings by breaking of thick and hard rock strata and proposed methods for disaster prevention and control. Wu et al^21^. used numerical simulation to study the surface deformation law of thick and hard rock stratum, and verified it through support pressure; Zhang et al^22^. used numerical simulation, theoretical analysis, and other methods to study the response characteristics of surface subsidence caused by the fracture of thick and hard rock stratum. The above research provides a lot of useful references for the study of the fracture response of thick and hard rock stratum, but there is a lack of research on the correlation between the movement evolution of thick and hard rock stratum and the occurrence of mining earthquakes and surface subsidence, making it difficult to comprehensively and deeply reveal the occurrence mechanisms of mining earthquakes and surface subsidence. This paper analyzes the disaster mechanism caused by the fracture evolution of thick and hard rock stratum in response to the special geological conditions in the Ordos region.

A large number of coal mines in China have typical Cretaceous thick hard sandstone deposits on the overlying strata. Therefore, it is necessary to study the fracture law and instability criteria of high-level hard-thick strata and conduct an in-depth analysis of the formation and instability process of overlying rock structures during large-scale coal mining. The research results have important guiding significance for the problems of strong rock pressure and surface subsidence in thick and hard rock stratum of coal mines. This paper takes the specific geological production conditions of hard-thick strata as the research background, and based on the Vlasov thick plate theory, establishes a mechanical model of the thick and hard key stratum thick plate. It reveals the fracture evolution process of hard-thick strata during the continuous mining process of the working face and verifies and analyzes it through high-energy mining earthquake events and surface subsidence response laws. The research results are applicable to mining areas in China, such as Inner Mongolia and Shandong, where high level thick and hard rock formations occur. At the same time, hard-thick strata is widely distributed in areas such as Shaanxi, Inner Mongolia, and Shandong in China, the research results of this paper have important guiding significance for the prevention and control of dynamic disasters caused by large-scale mining of thick and hard rock formations under similar geological conditions, as well as safe and efficient coal mining.

## Engineering background

Shilawusu coal mine is located in the territory of Ordos City, and its administrative division is under the jurisdiction of Iginhoro Banner of Ordos City. Its geographical coordinates are 109°35′00″–109°41′45″ East longitude and 38°58′52″–39°03′00″ North latitude. The minefield is a trapezoid, with a width of 7.35 km from north to south and an average length of about 9.40 km from east to west, covering an area of about 70.644 km^2^. The well field has typical plateau accumulation type sand dune landform features, and is a desert to semi-desert area. The main mining area is the 2–2# coal seam, with a thickness of 8.90–10.69 m and a burial depth of 644.60–672.78 m. The mining speed of the working face is 5–7 m/d, with a strike length of 2337.2–3238.7 m and a dip length of 268–289 m; The roof of the coal seam is mainly composed of fine sandstone and siltstone, with a hard sandstone formation with a thickness of 240–270 m occurring at a distance of 250–340 m above the coal seam. The mine has a design capacity of 10 million t/a, an approved capacity of 8 million t/a, recoverable reserves of 1536.25Mt, and a service life of 129a. The mine is divided into 3 mining areas, namely: 221 mining areas, 222 mining areas, and 223 mining areas.

**Figure 1:**
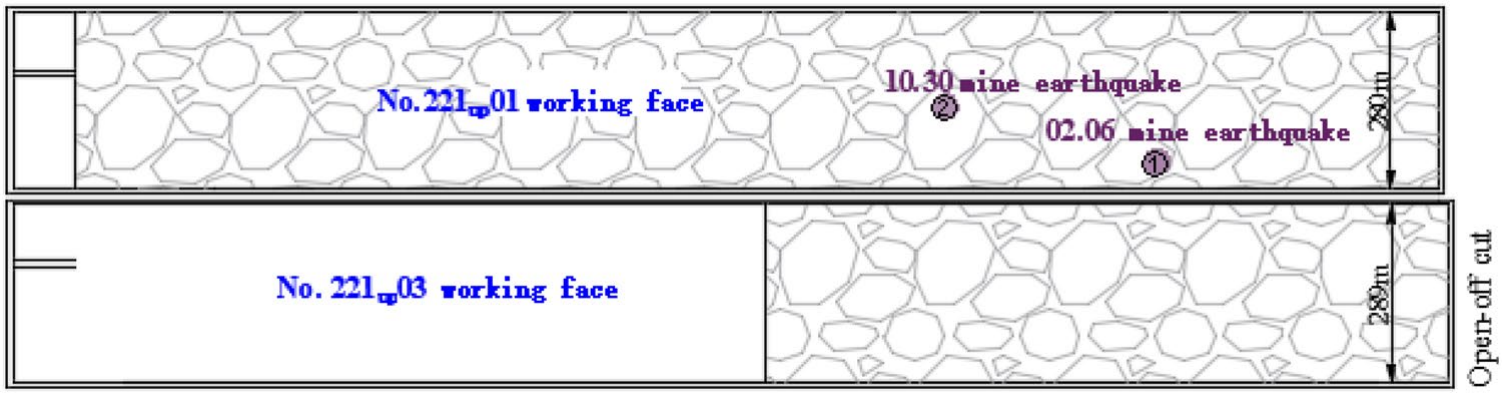
The layout of 221 Upper 01 and 221 Upper 03 working faces in Shilawusu.

The mine is currently mining the 221 mining area, with 2 working faces: 221 upper 08(now out of production) and 221 Upper 03. The north side of 221 on 03 working face is open-off cut, the south side is auxiliary transport roadway 1, belt transport roadway, return air roadway, and auxiliary transport roadway 2, and the east side is 221 on 05 comprehensive mining face (not mined), and the west is 221 upper fully mechanized mining face goaf. The elevation of the coal seam ranges from + 685.2 m to + 700.6 m, with an average of + 692.9 m. The surface elevation above the working face ranges from + 1354.7 to + 1396.7 m, with an average of + 1378.4 m. The average feed is 1273.4 m. The inclined length of 221 upper 01 working face is 280 m, the inclined length of 221 upper 03 working face is 289 m, and the strike length of 221 upper 03 working face is 2337 m. The width of the coal pillar is 5 m, as shown in Fig. 1.

Two large-energy strong mining earthquakes occurred during mining at the 221 upper 03 working face of the Shilawusu coal mine. On February 6, 2021, at 6:29:21, a vibration event with an energy of 4.78E + 06 J was monitored by the microseismic monitoring system of the Shilawusu coal mine. At this time 221 upper 03 working face has been advanced to mining 837 m, the location of the mine earthquake occurred 371 m behind the working face, 466 m from the original open-off cut of 221 upper 03 working face. On October 30, 2021, at 13:17:55, during the pushing process of 221 upper 03 working face of Shilawusu coal mine, a vibration event with energy of 4.15E + 05 J occurred in the mining goaf of 221 upper 01 working face, 815 m from the original open-off cut of 221 upper 03 working face, 338 m behind the coal wall of 221 upper 03 working face. At this time 221 upper 03 working face had been advanced to 1153 m, and the location of the large energy mine earthquake event is shown in Fig. 1. Both large energy events caused great concern to the local government, and the coal mine was ordered to stop production for rectification, which seriously affected the safe and efficient mining of coal.

**Figure 2:**
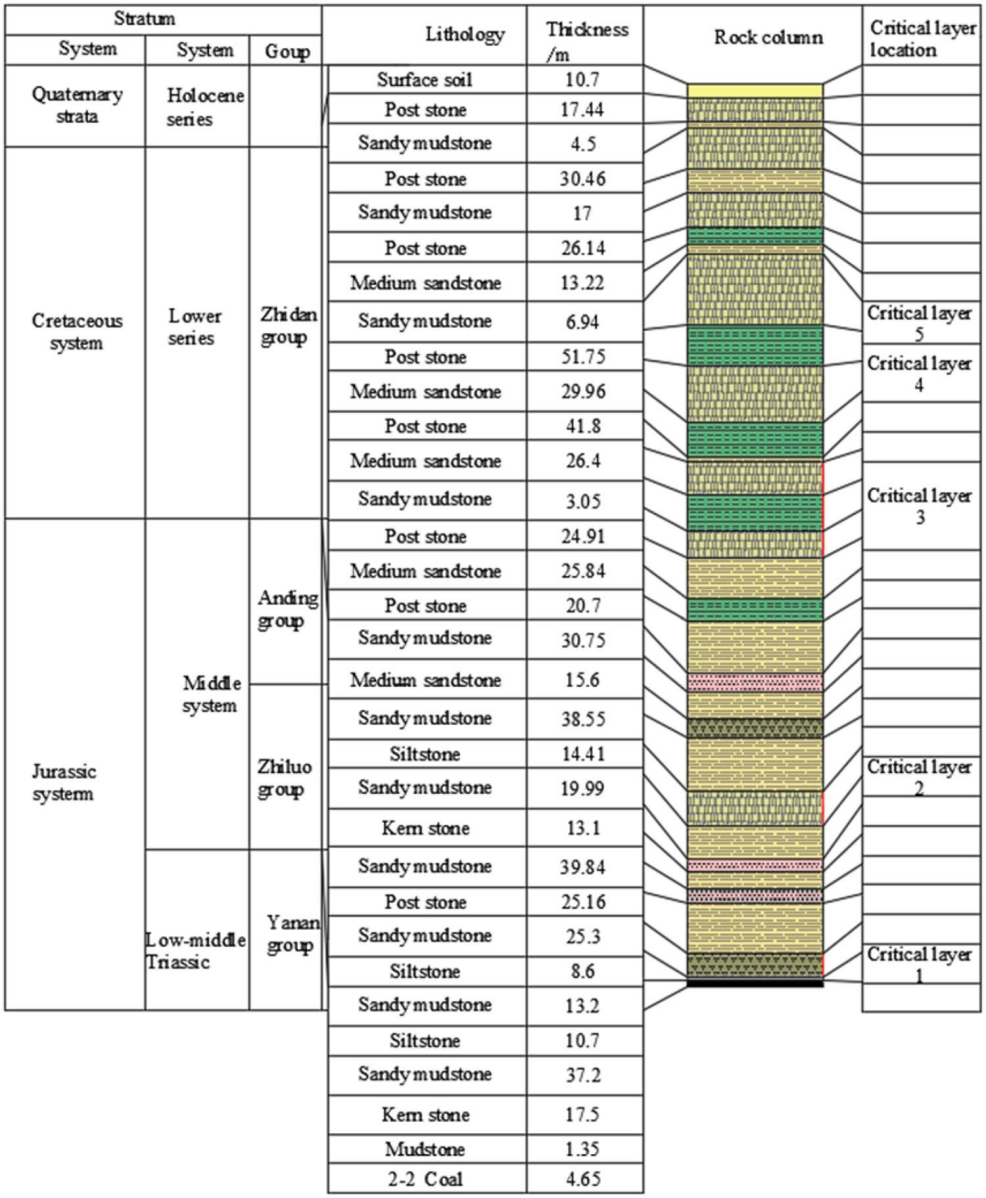
Geological column diagram of the 221 upper 03 working face of Shilawusu.

Figure 2 shows the geological bar chart of the 221 upper 03working face. From the theoretical calculation of key strata, it can be seen that above the working face of 221 Upper 03, there are five key strata. Key strata 1 and 2 are both located in the Jurassic stratum, with thicknesses of 17.5 m and 25.16 m respectively. Key strata 3, 4, and 5 are all located in the chalk strata. The chalk strata are mainly medium and fine-grained sandstones, which are hard, high strength, good integrity, far from the working face, and not easy to break, and are the main rock strata for strong mining earthquakes. Therefore, it is extremely necessary and urgent to carry out targeted research on the breaking pattern of the Cretaceous strata.

## Mechanical analysis of hard-thick strata

It is known from the theory of mine rock movement that in the process of coal mining, as the mining area continues to increase, the overlying rock strata in the mining area continuously collapse from the bottom up, and the fissures gradually develop upward and stop at the bottom of the thick hard key strata, forming the separation space^23^. As the mining area continues to increase, the separation space gradually increases, and the thick hard key strata gradually overhang and accumulate energy. When the breakage limit is reached, the breakage of the hard-thick strata will release a large amount of accumulated elastic energy to affect the overburden movement of the whole mining site, and even produce events such as strong surface subsidence and large energy mine earthquakes, which will threaten the safe and efficient production of the mine. It should be noted that during the mining phase of working face 03 on 221, due to the limited extent of the extraction area, the breaking movement mainly occurred in the key strata 3 within the cretaceous system. Therefore, the hard-thick strata in this paper refer to the key strata 3, whose thickness is 71 m and 311 m from the coal seam.

Due to the large thickness and high strength of key strata 3 in the stratum, a large overhanging roof will be formed before the initial breakage, and the analysis of the breakage law by using beam theory or thin plate theory calculation will produce a large deviation. From the theory of plate structure^24^, it is known that when the ratio between the thickness of the rock strata and the minimum characteristic size of the broken section is less than 1/5, the relevant theory of the elastic thin plate can be used to solve the problem, but when the ratio between the thickness of the rock strata and the minimum characteristic size of the broken section is greater than 1/5, the relevant theory of the thick plate should be used to solve the problem. According to the stratigraphic conditions of Shilawusu and the mining experience of the neighboring mines, it is known that the thickness of the hard-thick strata in the overlying cretaceous strata on 221 Upper 01 and 221 Upper 03 workings is 71 m. In this paper, the problem of breaking the hard-thick strata of the cretaceous strata (key stratum 3) is treated as a thick plate structure problem.

### Mechanics modeling

There are no obvious cutting faults around working face 221 upper 01 and 221 upper 03, and this paper mainly considers the initial breaking of the hard-thick strata of the cretaceous system. In the initial stage of rock movement, the main key strata are embedded in the rock body and supported by the lower strata, forming the boundary state of solid support all around. When the departure strata gradually become larger, the lower supporting rock strata of the thick hard rock stratum changes and gradually transforms into the surrounding simple support state. As a result, the broken boundary of the hard-thick strata is regarded as the surrounding simple support boundary. And the following assumptions are made^25^: (1) The hard-thick strata belong to the category of linear elastic deformation before breaking, which is in accordance with Hooke’s law. (2) The hard-thick strata is considered continuous, consistent with the continuity assumption. (3) The hard-thick strata are straight lines perpendicular to the middle surface before deformation and bend into a parabola after deformation. (4) The hard-thick strata are in a generalized plane stress state and ignore the effect of transverse strain. (5) The deflection and stress of the hard-thick strata are distributed linearly and regularly along the direction of flat thickness. What needs illustration is that the mechanical analysis of hard-thick strata is based on continuum linear elastic mechanics, but when the hard-thick strata is driven close to breaking point, the use of continuum linear elasticity for analysis has great limitations has great limitations, and we will continue to improve the model in the future study.

**Figure 3:**
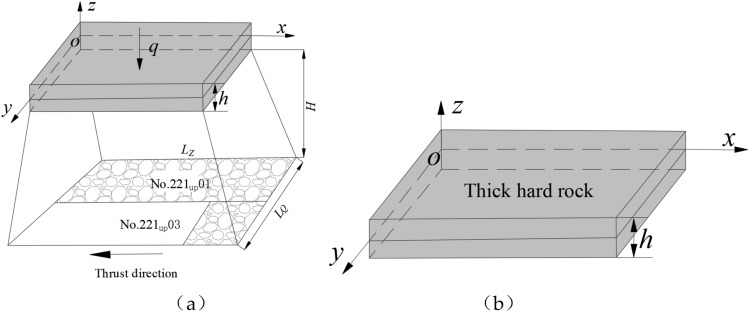
Mechanical model of thick plate.

Based on the above assumptions, the mechanical model of the thick plate with solid support all around is established with the middle surface of the main key stratum of the cretaceous sandstone as the reference surface, as shown in Fig. 3. Where: the x-axis is the direction of workface strike advance, the y-axis is the direction of workface inclination, the z-axis is the vertical direction, the origin O is located at the intersection of the three axes in the middle face of the thick plate, h is the thickness of the thick hard rock stratum, a is the strike overhang length when the rock stratum is broken, b is the inclination overhang length when the rock stratum is broken, q(x, y) is the uniform load on the hard-thick strata, H is the distance between the main key strata and the coal seam, L_Z_ and L_Q_ is the advance length of the workface along the strike and inclination when broken.

### Fracture mechanics analysis of hard-thick rock strata

Considering the thick and hard main key strata assignment conditions and the complexity of bending motion, a simplified Vlasov theory^25^ is used in this paper for the solution.

In general, the length and width of rock stratum are much greater than their thickness, so the stratum can be considered as rectangular thin plates for analysis. However, in practical engineering, due to the large thickness of high thick and hard rock stratum, the ratio of length, width, and thickness does not comply with the thin plate theory. If the thin plate theory is still used and the lateral shear deformation of the rock stratum is ignored, the research results will deviate from engineering practice. Therefore, some scholars have started to use the thick plate theory for research. Considering the occurrence conditions of thick and hard key stratum and the complexity of bending motion, this paper adopts the simplified Vlasov theory^25^ to solve.

Based on the Vlasov theory, the displacement function and stress component are simplified to obtain a set of differential equations for thick plate bending expressed in terms of deflection and rotation angle:

1 $$\begin{gathered} \nabla^{2} \psi_{{\text{x}}} + \frac{1 + \nu }{2}\frac{\partial }{\partial y}\left( {\frac{{\partial \psi_{{\text{y}}} }}{\partial x} - \frac{{\partial \psi_{{\text{x}}} }}{\partial y}} \right) + \frac{1}{4}\frac{\partial }{\partial x}\left( {\nabla^{2} \omega } \right) = \frac{5Gh}{{6D}}\left( {\psi_{{\text{x}}} - \frac{\partial \omega }{{\partial x}}} \right) \\ \nabla^{2} \psi_{{\text{y}}} + \frac{1 + \nu }{2}\frac{\partial }{\partial x}\left( {\frac{{\partial \psi_{x} }}{\partial y} - \frac{{\partial \psi_{y} }}{\partial x}} \right) + \frac{1}{4}\frac{\partial }{\partial y}\left( {\nabla^{2} \omega } \right) = \frac{5Gh}{{6D}}\left( {\psi_{y} - \frac{\partial \omega }{{\partial y}}} \right) \\ \frac{{\partial \psi_{x} }}{\partial x} + \frac{{\partial \psi_{y} }}{\partial y} - \nabla^{2} \omega = \frac{3}{2Gh}q\left( {x,y} \right) \\ \end{gathered}$$

where, D is the flexural stiffness; E is the modulus of elasticity; h rock thickness; *Ψ*_*x*_, *Ψ*_*y*_ are the turning angles of x, and y, respectively; ν is the Poisson's ratio; ω is the deflection; G is the shear deformation modulus; q is the uniform load.

The boundary conditions of the four-perimeter solid support plate structure are as follows:

2 $$\begin{gathered} \omega \left| {_{x = 0,x = a} } \right. = 0,\psi_{y} \left| {_{x = 0,x = a} } \right. = 0,M_{y} \left| {_{x = 0,x = a} } \right. = 0 \\ \omega \left| {_{y = 0,y = b} } \right. = 0,\psi_{x} \left| {_{y = 0,y = b} } \right. = 0,M_{y} \left| {_{y = 0,y = b} } \right. = 0 \\ \end{gathered}$$

Using Eq. (2), the expressions of internal force and internal moment can be obtained:

3 $$\begin{gathered} M_{x} = - \frac{D}{5}\left[ {4\left( {\frac{{\partial \psi_{x} }}{\partial x} + \nu \frac{{\partial \psi_{y} }}{\partial y}} \right) + \left( {\frac{{\partial^{2} \omega }}{{\partial x^{2} }} + \nu \frac{{\partial^{2} \omega }}{{\partial y^{2} }}} \right)} \right] \hfill \\ M_{y} = - \frac{D}{5}\left[ {4\left( {\frac{{\partial \psi_{y} }}{\partial y} + \nu \frac{{\partial \psi_{x} }}{\partial x}} \right) + \left( {\frac{{\partial^{2} \omega }}{{\partial y^{2} }} + \nu \frac{{\partial^{2} \omega }}{{\partial x^{2} }}} \right)} \right] \hfill \\ M_{xy} = - \frac{{D\left( {1 - \nu } \right)}}{5}\left[ {2\left( {\frac{{\partial \psi_{x} }}{\partial y} + \frac{{\partial \psi_{y} }}{\partial x}} \right) + \frac{{\partial^{2} \omega }}{\partial x\partial y}} \right] \hfill \\ Q_{x} = \frac{2}{3}Gh\left( {\frac{\partial \omega }{{\partial x}} - \psi_{x} } \right),Q_{y} = \frac{2}{3}Gh\left( {\frac{\partial \omega }{{\partial y}} - \psi_{y} } \right) \hfill \\ \end{gathered}$$

The differential equilibrium equation can be written as:

4 $$\begin{gathered} \frac{2D}{5}\left[ {\left( {1 - \nu } \right)\nabla^{2} \psi_{x} + \left( {1 + \nu } \right)\frac{\partial \Phi }{{\partial x}} + \frac{1}{2}\frac{\partial }{\partial x}\left( {\nabla^{2} \omega } \right)} \right] + \frac{2}{3}Gh\left( {\frac{\partial \omega }{{\partial x}} - \psi_{x} } \right) = 0 \\ \frac{2D}{5}\left[ {\left( {1 - \nu } \right)\nabla^{2} \psi_{y} + \left( {1 + \nu } \right)\frac{\partial \Phi }{{\partial y}} + \frac{1}{2}\frac{\partial }{\partial y}\left( {\nabla^{2} \omega } \right)} \right] + \frac{2}{3}Gh\left( {\frac{\partial \omega }{{\partial y}} - \psi_{y} } \right) = 0 \\ \frac{2}{3}Gh\left( {\nabla^{2} \omega - \Phi } \right) + q\left( {x,y} \right) = 0 \\ \Phi = \frac{{\partial \psi_{x} }}{\partial x} + \frac{{\partial \psi_{y} }}{\partial y} \\ \end{gathered}$$

Let the deflection and turning angle functions be:

5 $$\begin{gathered} \omega = \sum\limits_{m = 1}^{\infty } {\sum\limits_{n = 1}^{\infty } {A_{mn} } } \sin \frac{m\pi x}{a}\sin \frac{n\pi y}{b} \hfill \\ \psi_{x} = \sum\limits_{m = 1}^{\infty } {\sum\limits_{n = 1}^{\infty } {B_{mn} } } \cos \frac{m\pi x}{a}\sin \frac{n\pi y}{b} \hfill \\ \psi_{y} = \sum\limits_{m = 1}^{\infty } {\sum\limits_{n = 1}^{\infty } {C_{mn} } } \sin \frac{m\pi x}{a}\cos \frac{n\pi y}{b} \hfill \\ \end{gathered}$$

The load is also spread into a double delta level:

6 $$q(x,y) = \sum\limits_{m = 1}^{\infty } {\sum\limits_{n = 1}^{\infty } {q_{mn} } } \sin \frac{m\pi x}{a}\sin \frac{n\pi y}{b}$$

Substituting (5) and (6) into (3) gives:

7 $$\begin{gathered} A_{mn} = \left\{ {1 + \frac{{6D\pi^{2} }}{5Gh}\left[ {\left( \frac{m}{a} \right)^{2} + \left( \frac{n}{b} \right)^{2} } \right]} \right\} \cdot {{q_{mn} } \mathord{\left/ {\vphantom {{q_{mn} } {D\pi^{4} \left[ {\left( \frac{m}{a} \right)^{2} + \left( \frac{n}{b} \right)^{2} } \right]^{2} }}} \right. \kern-0pt} {D\pi^{4} \left[ {\left( \frac{m}{a} \right)^{2} + \left( \frac{n}{b} \right)^{2} } \right]^{2} }} \hfill \\ B_{mn} = \left\{ {1 - \frac{{3D\pi^{2} }}{10Gh}\left[ {\left( \frac{m}{a} \right)^{2} + \left( \frac{n}{b} \right)^{2} } \right]} \right\} \cdot {{mq_{mn} } \mathord{\left/ {\vphantom {{mq_{mn} } {aD\pi^{3} \left[ {\left( \frac{m}{a} \right)^{2} + \left( \frac{n}{b} \right)^{2} } \right]^{2} }}} \right. \kern-0pt} {aD\pi^{3} \left[ {\left( \frac{m}{a} \right)^{2} + \left( \frac{n}{b} \right)^{2} } \right]^{2} }} \hfill \\ C_{mn} = \left\{ {1 - \frac{{3D\pi^{2} }}{10Gh}\left[ {\left( \frac{m}{a} \right)^{2} + \left( \frac{n}{b} \right)^{2} } \right]} \right\} \cdot {{nq_{mn} } \mathord{\left/ {\vphantom {{nq_{mn} } {aD\pi^{3} \left[ {\left( \frac{m}{a} \right)^{2} + \left( \frac{n}{b} \right)^{2} } \right]^{2} }}} \right. \kern-0pt} {aD\pi^{3} \left[ {\left( \frac{m}{a} \right)^{2} + \left( \frac{n}{b} \right)^{2} } \right]^{2} }} \hfill \\ \end{gathered}$$

Expanding the system of equations Amn, Bmn, and Cmn levels to one level (m = n = 1), gives:

8 $$\begin{gathered} A_{11} = \left[ {1 + \frac{{6D\pi^{2} }}{5Gh}\left( {\frac{1}{{a^{2} }} + \frac{1}{{b^{2} }}} \right)} \right] \times \frac{{q_{11} }}{{D\pi^{4} \left( {\frac{1}{{a^{2} }} + \frac{1}{{b^{2} }}} \right)^{2} }} \hfill \\ B_{11} = C_{11} = \left[ {1 - \frac{{3D\pi^{2} }}{10Gh}\left( {\frac{1}{{a^{2} }} + \frac{1}{{b^{2} }}} \right)} \right] \times \frac{{q_{11} }}{{aD\pi^{3} \left( {\frac{1}{{a^{2} }} + \frac{1}{{b^{2} }}} \right)^{2} }} \hfill \\ \end{gathered}$$

Substituting (5), (6), and (8) into (3), gives M_x_, M_y_:

9 $$\begin{gathered} M_{x} = \frac{D}{5}\left[ {\frac{{q_{11} \left( {\frac{5}{{a^{2} }} + \frac{4\nu }{{ab}} + \frac{\nu }{{b^{2} }}} \right)}}{{D\pi^{2} \left( {\frac{1}{{a^{2} }} + \frac{1}{{b^{2} }}} \right)^{2} }} + \frac{{6q_{11} \nu \left( {\frac{1}{{b^{2} }} - \frac{1}{ab}} \right)}}{{5Gh\left( {\frac{1}{{a^{2} }} + \frac{1}{{b^{2} }}} \right)}}} \right] \times \sin \frac{\pi x}{a} \times \sin \frac{\pi y}{b} \hfill \\ M_{y} = \frac{D}{5}\left[ {\frac{{q_{11} \left( {\frac{5}{{a^{2} }} + \frac{4\nu }{{ab}} + \frac{\nu }{{b^{2} }}} \right)}}{{D\pi^{2} \left( {\frac{1}{{a^{2} }} + \frac{1}{{b^{2} }}} \right)^{2} }} + \frac{{6q_{11} \nu \left( {\frac{1}{{a^{2} }} - \frac{1}{ab}} \right)}}{{5Gh\left( {\frac{1}{{a^{2} }} + \frac{1}{{b^{2} }}} \right)}}} \right] \times \sin \frac{\pi x}{a} \times \sin \frac{\pi y}{b} \hfill \\ \end{gathered}$$

Since Mx = My, the bending moment obtains its maximum value at x = a/2 and y = b/2, and we can make M_max_ = M_xmax_ to obtain:

10 $$M_{\max } = M_{x\max } = \frac{D}{5}\left[ {\frac{{q_{11} \left( {\frac{5}{{a^{2} }} + \frac{4\nu }{{ab}} + \frac{\nu }{{b^{2} }}} \right)}}{{D\pi^{2} \left( {\frac{1}{{a^{2} }} + \frac{1}{{b^{2} }}} \right)^{2} }} + \frac{{6q_{11} \nu \left( {\frac{1}{{b^{2} }} - \frac{1}{ab}} \right)}}{{5Gh\left( {\frac{1}{{a^{2} }} + \frac{1}{{b^{2} }}} \right)}}} \right]$$

The maximum tensile stress on the lower surface of the thick plate is:

11 $$\sigma_{\max } = \frac{{12M_{\max } }}{{h^{3} }}Z = \frac{{12M_{\max } }}{{h^{3} }} \times \frac{h}{2} = \frac{{6M_{\max } }}{{h^{2} }}$$

Considering that the tensile strength of hard-thick strata is the smallest and its damage form is mainly tensile damage when the stress value *σ*_*max*_ on the lower surface of the thick plate exceeds its tensile strength [*σ*_*s*_], the thick plate will undergo tensile damage, from which the critical mechanical conditions for the thick plate to break can be obtained as follows.

12 $$\sigma_{\max } = \left[ {\sigma_{s} } \right] = \frac{{6q_{11} \left( {\frac{5}{{a^{2} }} + \frac{4\nu }{{ab}} + \frac{\nu }{{b^{2} }}} \right)}}{{5\pi^{2} \left( {\frac{1}{{a^{2} }} + \frac{1}{{b^{2} }}} \right)^{2} h^{2} }} + \frac{{6q_{11} \nu \left( {\frac{1}{{b^{2} }} - \frac{1}{ab}} \right)}}{{25\left( {1 - \nu } \right)\left( {\frac{1}{{a^{2} }} + \frac{1}{{b^{2} }}} \right)}}$$

From Eq. (12), it can be seen that the breakage size characteristics of hard-thick strata will be influenced by the rock tensile strength [*σ*_*s*_], rock thickness h, overlying load q, Poisson's ratio *υ,* and other factors.

When the thick plate is infinitely long (b =  + ∞), the limit breaking distance of the thick plate can be obtained^15^:

13 $$a = \pi h\sqrt {\frac{{\left[ {\sigma_{s} } \right]}}{6q}}$$

When a = b, positive O-X breaking occurs in the thick plate rock formation, at which time the breaking distance is^15^:

14 $$a = \pi h\sqrt {\frac{{2\left[ {\sigma_{s} } \right]}}{3q(1 + \upsilon )}}$$

Based on the law of overlying rock rupture, combined with the engineering situation of thick hard rock seam and coal seam spacing, the size of the broken overhang of thick hard rock seam cannot be taken as the size of mining space, and the influence of rock seam rupture angle needs to be taken into account. So the corresponding relationship between the size of broken hard rock strata and the size of mining space is as follows.

15 $$\begin{gathered} a = L_{{\text{Z}}} - 2H\cot \alpha \hfill \\ b = L_{{\text{Q}}} - 2H\cot \alpha \hfill \\ \end{gathered}$$

where, α is the breaking angle of the rock stratum, L_Z_ is the length of the thick slab model at the working face along the strike direction, L_Q_ is the length of the thick plate model at the working face along the tendency direction, H is the distance from the working face to the lower surface of the hard-thick strata.

Combined with the specific engineering geological conditions, when the thick hard rock stratum is broken the overhang length a and tendency overhang length b along the strike can be obtained from Eqs. (12) to (14) respectively, and then the corresponding mining space strike and tendency scale L_Z_ and L_Q_ can be calculated from Eq. (15).

**Figure 4:**
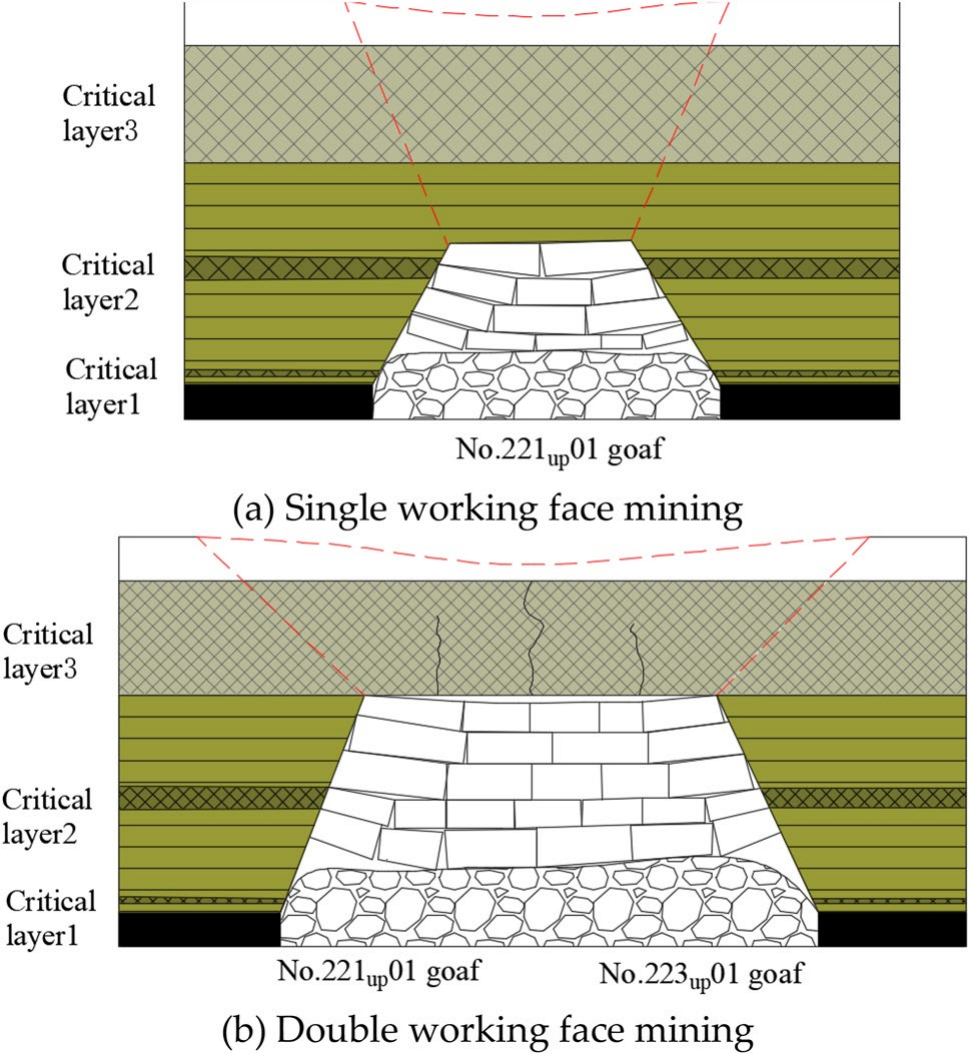
Single and double working face damage range.

Combining Eqs. (12) to (15) in a comprehensive analysis, it can be seen that under ideal conditions, breakage of a hard-thick strata will occur only when the breakage limit is reached along both strike and tendency dimensions. However, in actual engineering practice, the working face strike advance length is much greater than the inclination length. Under the condition of single face mining, even if the strike advancing distance reaches the breaking limit, if the inclined length is less than the breaking limit, no breaking will occur, and multiple faces need to be mined jointly so that the length of the inclined direction of the mining space also reaches the breaking limit before the hard-thick strata will break, as shown in Fig. 4.

## Fracture mechanism of hard-thick rock strata

According to the specific engineering geological conditions of the Shilawusu coal mine, the relevant parameters are substituted into Eqs. (12)–(15) for analysis and calculation, and the breakage distance law of key strata 3 can be analyzed. Based on the geological measurement data of the mine, it is known that [σs] = 3 MPa, υ = 0.25, α = 70°, h = 71 m, the thickness of the hard-thick strata and its upper overburden is 310 m, q = 8060kN/m^2^, and H = 311 m. From Eq. (12), (13), the limit breakage step is obtained for the limit state of the infinite length of thick slab a = 55.6 m. When a = b = 99.4 m, positive O–X breakage will occur in hard-thick strata^9^.

**Figure 5:**
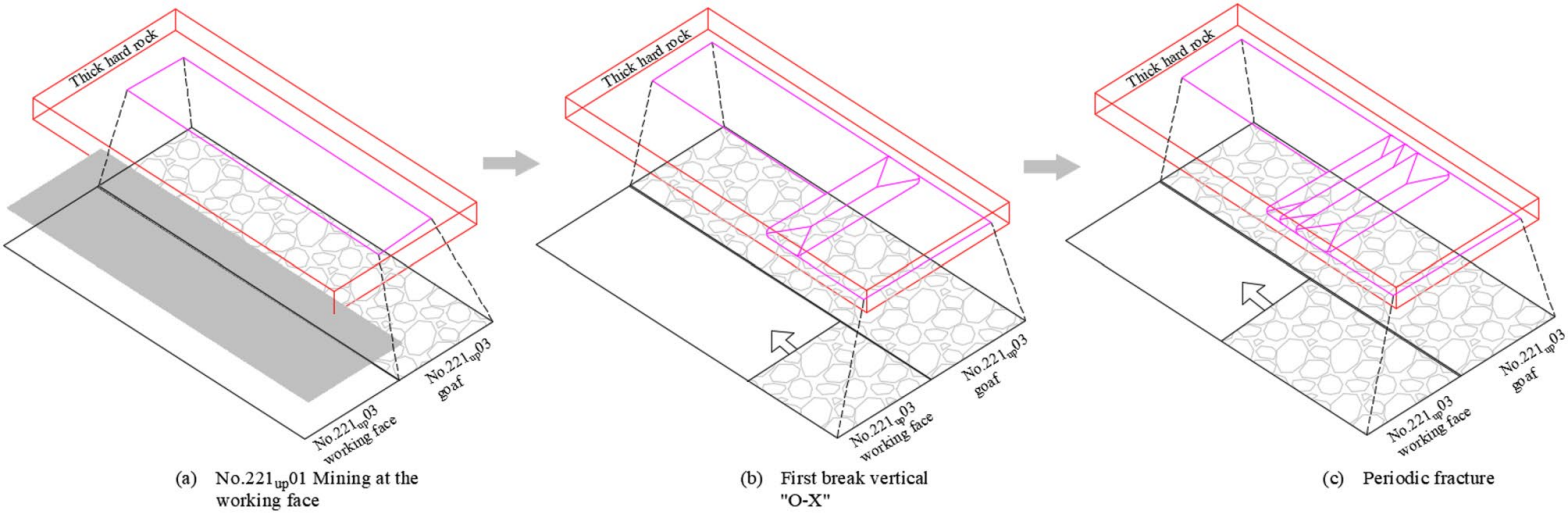
Evolutionary process of breakage of hard-thick strata.

1. During the mining period of the 221 upper 01 working face, L_Q_ = 280 m and L_Z_ = 2337 m, the strike length can be considered infinite. During this stage, the maximum overburden damage caused by the mining of the working face is about 1/2 of the short side length of the extraction area^26^, which is about 140 m. The overburden damage is small and fails to reach the cretaceous rock strata, and the key strata 3 maintains good stability. It is also known from Eq. (15) that b < 55.6 m when L_Q_ = 280 m, which is not enough to reach the limit breakage distance. Therefore, no breakage of the hard-thick strata will occur during mining on the 221 upper 01 working face.
2. In the early stage of the mining of the 221 upper 03 working face, as the working face advances, the overburden rupture height increases continuously, and when the mining is advanced to the double square stage, the overburden rupture height reaches the maximum, about 300 m. At this time, the overburden movement began to spread to the hard-thick strata of the cretaceous, and the lower part of key strata 3 began to appear as a separation space and began to overhang the top. The inclined length L_Q_ of the two working faces of the 221 upper 01 and of the 221 upper 03 during this stage is 574 m, and the corresponding inclined overhang length of the key stratum b = 348 m > 55.6 m, which meets the breaking requirements. At this time, the hard-thick strata conform to the thick plate structure, and the application of the thick plate theory yields the overhang length of the hard-thick strata towards 56 m, that is, the hard-thick strata start to overhang the top. When the working face continued to advance 282 m, the hard-thick strata broke. The inclined overhang length b = 348 m > a = 99.4 m when the hard-thick strata is broken, so it is decided that the rock strata will be broken in a vertical O–X direction.
3. After the initial breakage of the hard-thick strata, periodic breakage of the hard-thick strata will occur as the 221 upper 03 working face continues to advance. According to the calculation of the “cantilever beam” structure^27,28^, the periodic breakage distance of the hard-thick strata is 25 m. In other words, the periodic breakage of the hard-thick strata will occur when the working face advances at least 138 m, and the cycle will continue until the end of the working face, as shown in Fig. 5.

## Verification of microseismic activity

Shilawusu coal mine is currently equipped with the SOS microseismic monitoring system to monitor vibrations with an energy of 102 J or more, which is capable of localization and energy calculation, with no less than six stations around the 221 upper 03 working face for effective monitoring. Prior to the mining of the 221 Upper 03 working face on the north flank of the 221 mining area in Shilawusu, the 221 Upper 01 working face had been retrieved. Small energy microseismic events were frequent during the mining of the 221 upper 03 working face, and two large energy mine earthquakes occurred. Therefore, it is important to study and analyze the law of occurrence of large energy events to prevent coal mine safety and carry out relevant management work in a targeted manner.

### Microseismic activity characteristics

The 221 upper 03 working face started mining in June 2020 and has now advanced 1273.4 m. The 221 upper 03 working face stopped production on August 29, 2021, and resumed production on October 21, 2021, at which time the 221 upper 03 working face was advanced to about 1100 m, close to the double working face squared area.

**Figure 6:**
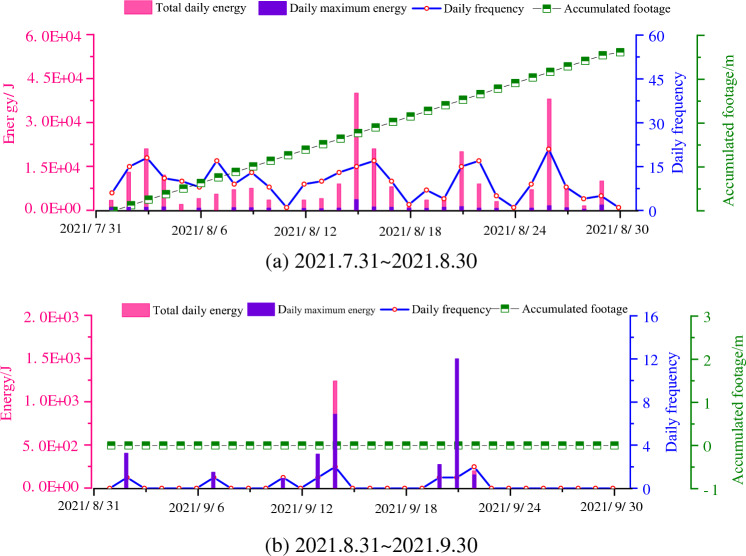
Relationship between mining and microseismic activity on the 221 upper 03 working face.

From Fig. 6a, it can be seen that before the working face was stopped, the working face was always mined at a uniform speed. The microseismic events were more active, and the microseismic frequency and the total daily energy showed periodic changes. The daily microseismic frequency was below 20 times, and the total daily energy was below 4.5 × 104 J. As can be seen from Fig. 6b, after the shutdown of the 221 upper 03 working face, the magnitude and frequency of microseismic energy decreased substantially, and even no microseismic occurred.

**Figure 7:**
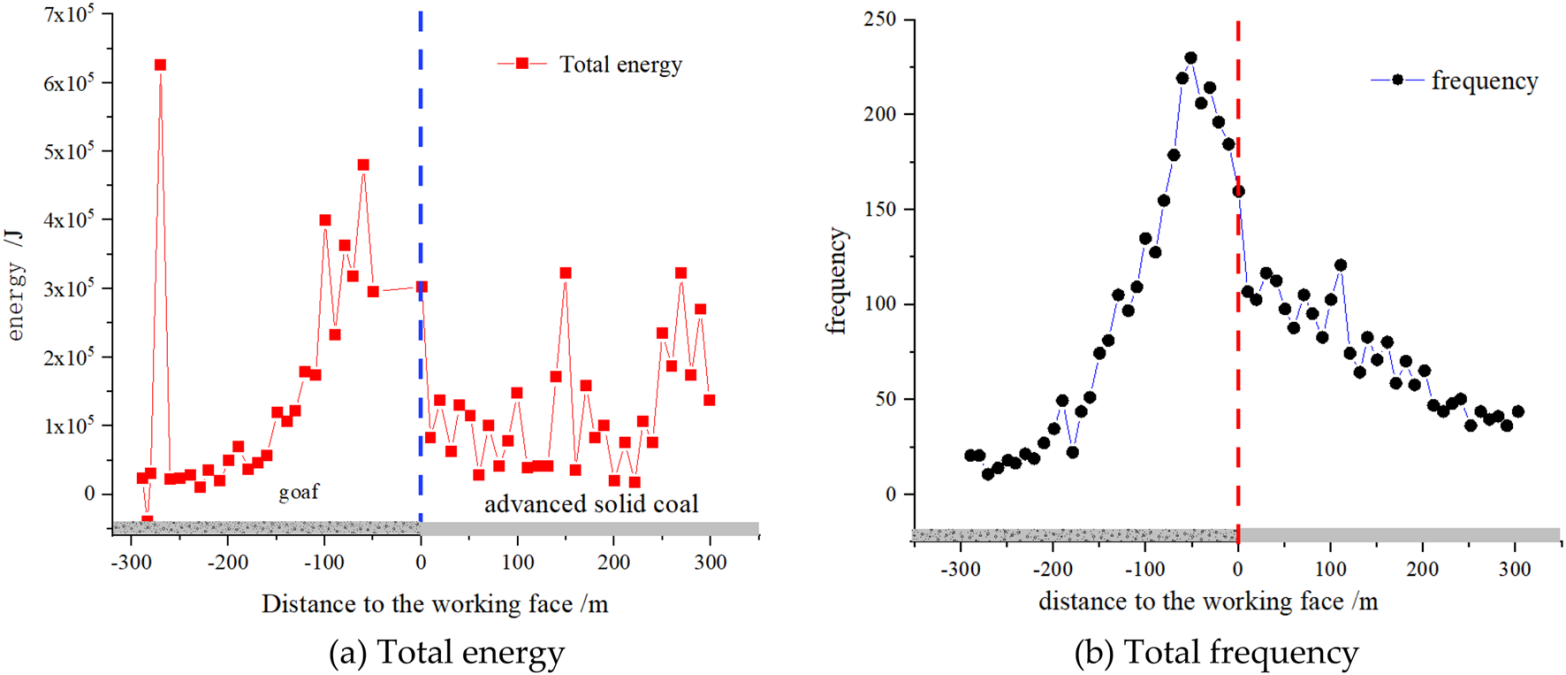
Distribution pattern of microseisms before the first square error.

**Figure 8:**
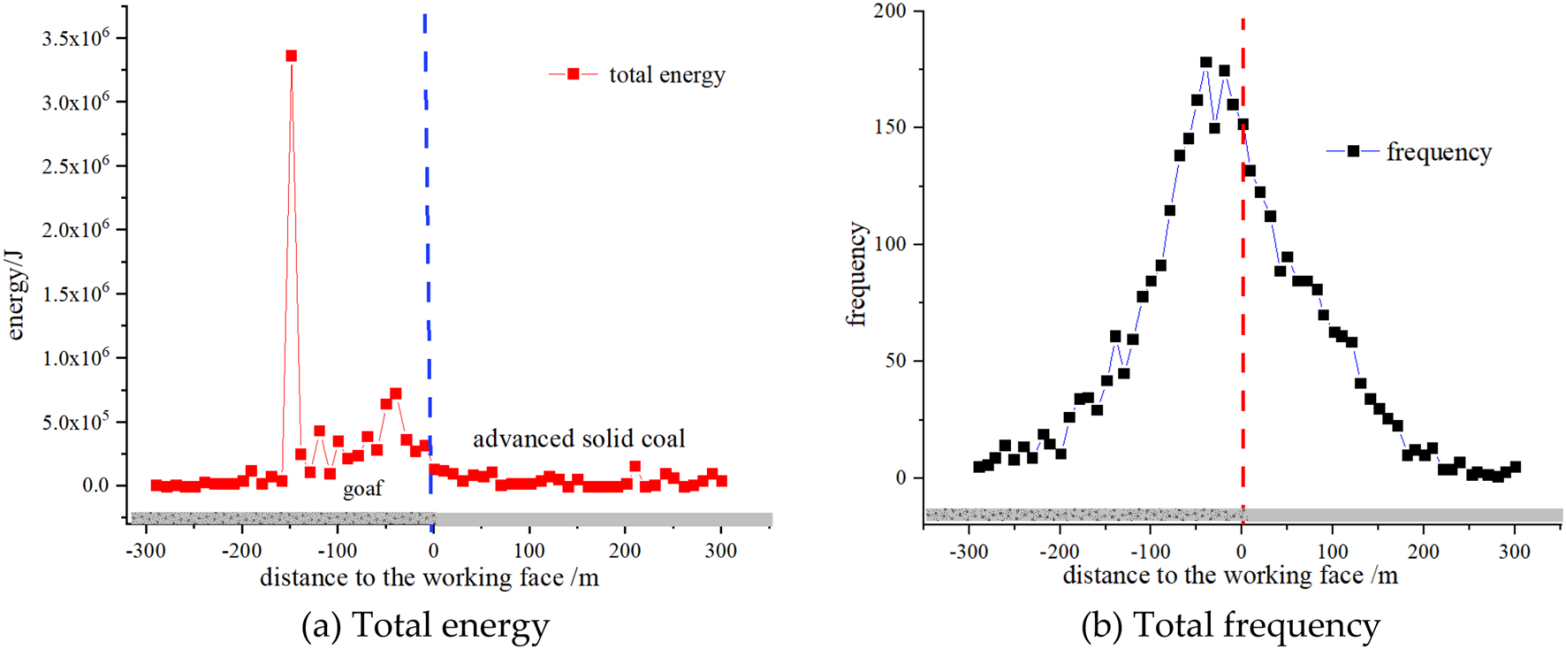
Distribution pattern of microseisms after the first square error.

Further analysis of Figs. 7 and 8 shows that when the mining distance of the working face does not reach the first square, microseismic activity is mainly concentrated on one side of the goaf. Within a range of 60 m behind the working face, microseismic events are relatively concentrated, with a total energy of up to 5.5 × 10^5^ J. The leading area of the working face is mainly characterized by high-frequency and low-energy microseismic events. As the working face is pushed to the square area near the double working face, microseismic activity still mainly occurs behind the goaf, and the range of dynamic load disturbance gradually narrows, resulting in a significant increase in total energy. Therefore, it can be seen that the occurrence of microseismic events is mainly caused by the fracture of thick and hard rock stratums above the goaf roof. Due to the gradual increase in the scope of the goaf, there is a large movement space in the overlying rock structure, resulting in the occurrence of microseismic events on one side of the goaf in the form of low frequency and high energy.

### Analysis of high-energy mine earthquake activity

After the first square footage, a total of 7 high-energy mine tremors occurred in the Shilawusu Coal Mine, which had a certain impact on the safety production of the coal mine. A total of three high-energy mining earthquakes occurred in the north wing working face, of which two occurred in the 221-03 working face and one occurred in the 221-17 working face. There have been 4 occurrences at the southern-wing working face, mainly in the goaf of the 221-08 working face and the 221-06A working face.

By summarizing and analyzing the historical mining earthquake locations and monitoring data of this mine, it is clear that the historical mining earthquake characteristics of the south and north wings of this mine are: after the mining face enters the goaf, especially in the single face and double face stages, mining earthquakes are prone to occur, which is mainly caused by the large-scale initial breaking of the main key stratum.

**Figure 9:**
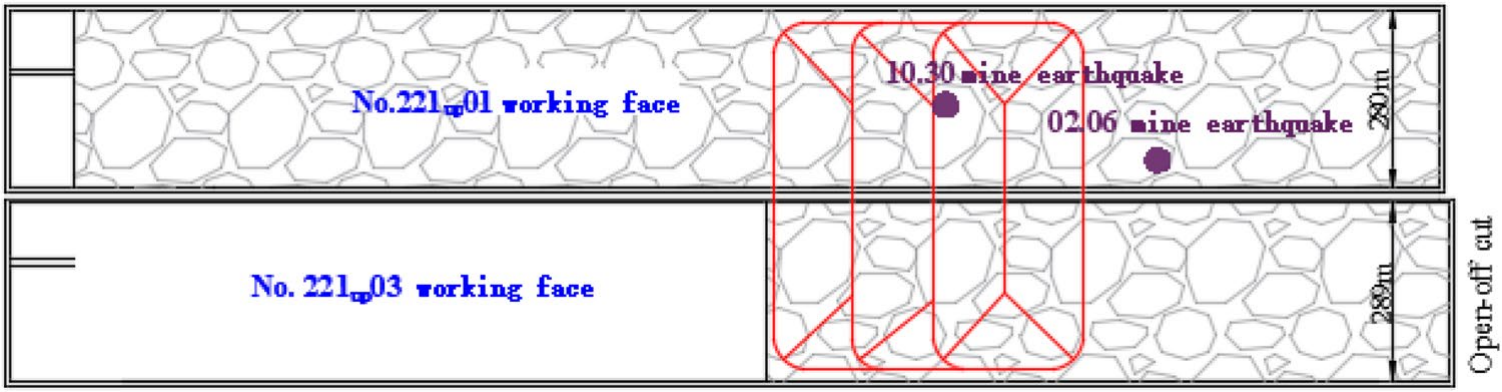
Distribution of large energy mine seismic during mining at the 221 upper 01 working face.

The first large energy mine earthquake event occurred on February 6, 2021, during the mining period on the 221 Upper 03 working face, which was mined to 837 m at this time. On October 30, 2021, the second large-energy mine earthquake event occurred when the working face was mined to 1153 m. There is a strong correlation between the two large energy mine earthquake events and the vertical “O–X” breakage of the thick plate, as shown in Fig. 9.

1. During the mining period of the 221 upper 01 working face, both sides are solid coal, and the rock damage range is small. The overlying rock did not destroy the collapse, and no large energy mine seismic event occurred. When mining reached the 221 upper 01 working face, one side is solid coal, the other side is the 221 upper 01 working face goaf area. Two working faces form a large area of mining void area, the overlying surrounding rocks on the two working faces occur synergistic effect, the overlying rocks damage range becomes larger, and hard-thick strata begins to separate and damage.
2. At the time of the “2.6” earthquake, the working face was mined to 837 m, and the calculation result of the thick plate theory shows that the first vertical “O–X” break of the hard-thick strata occurred when the hard-thick strata was advanced to 856 m from 574 m away from the open-off cut, and the two data are close to each other. Therefore, the “2.6” earthquake can indirectly prove the correctness of the initial breakage theory. At the time of the “10.30” earthquake, the working face was mined to 1153 m. Using the cantilever beam theory, we can get that the cycle breakage distance is 138 m, which is 1132 m from the working face when two cycle breakages occurred in the hard-thick strata, which is close to 1153 m from the open-off cut when the “10.30” earthquake occurred and proves the correctness of the cycle breakage theory calculation of the hard-thick strata.

## Response characteristics of surface subsidence

**Figure 10:**
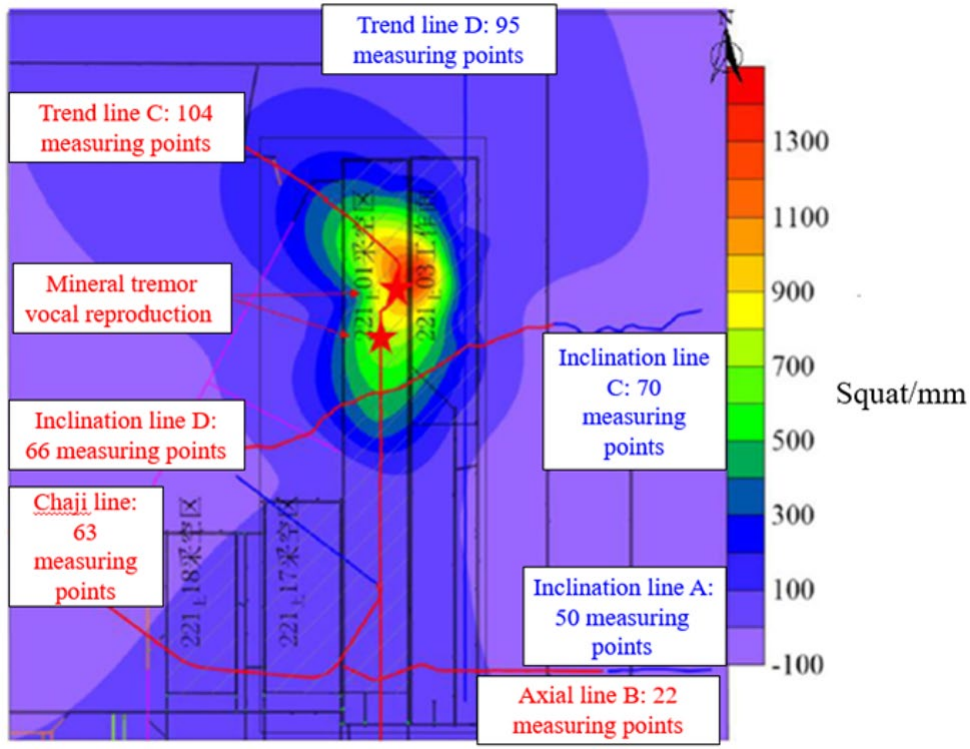
Amount of surface subsidence.

In order to verify the correctness of the fracture evolution process of thick and hard rock stratum, the surface subsidence during the push mining process of Shilawusu Coal Mine was monitored and analyzed, and the surface response characteristics induced by the fracture evolution of the thick and hard main key stratum were analyzed. The observation lines of surface subsidence observation points were arranged on the ground surface of the 221 upper 01 and the 221 upper 01 working faces, as shown in Fig. 10.

From Fig. 10, it can be seen that the value of the surface settlement is small, about 200 mm, when only the 221 upper 01 working face is mined. The surface settlement was not sufficient at this time, indicating that the overburden damage was small and the hard-thick strata (key strata 3) did not break during mining at the 221 upper 01 working face. When the mining at the 221 upper 01 working face began, the surface sinkage at the 221 upper 01 working face and the 221 upper 03 working face mining area increased significantly, and the surface sinkage was the largest at measurement point D80 on the inclination line between the 221 upper 01 working face mining area and the 221 upper 03 working face mining area, with the maximum sinkage amount reaching 1397 mm.

When the 221 upper 03 working face is mined, the value of surface subsidence in the 221 upper 01 and 221 upper 03 mining goaf area increases significantly. The influence of the large mining goaf area formed by the 221 upper 01 and 221 upper 03 double working on the rupture range of the upper overlying rock is further increased and affects the hard-thick strata, which leads to a significant increase in the amount of surface subsidence. With the continued mining of the 221 upper 03 working face, the area of the mining void area becomes larger, the overlying rock destruction becomes larger and more fully destroyed, and the amount of subsidence increases as the working face advances. Moreover, the locations of the two high-energy mining earthquakes coincide highly with the area of maximum surface subsidence, indicating that the high-energy mining earthquakes are closely related to the breakage of the overlying rock strata in the mining area.

## Conclusions

1. The hard-thick strata in the cretaceous system will not be broken during the advancing and mining process of the 221 upper 03 working face of the Shilawusu coal mine. And before the advancing and mining of the 221 upper 03 working face reached “double square”, no breakage occurred in the lower part of the hard-thick strata because no separated space was formed.
2. When the 221 upper 03 working face was advanced to about 856 m, vertical “O–X” breakage occurred in the hard-thick strata. As the working face continues to advance 138 m, periodic breakage will continue to occur in the hard-thick strata. No large energy mine earthquake event has occurred during mining at 221 upper 03 working face, and the amount of surface subsidence is approximately 200 mm.
3. In the mining at 221 upper 03 working face, two large energy mining earthquakes occurred at 837 m, and 1153 m away from the open-off cut, respectively, and the maximum amount of surface subsidence increased to 1397 mm, which basically accords with the results of the first and periodic breaks calculated by theory.

## Data Availability

Data associated with this research are available and can be obtained by contacting the corresponding author upon reasonable request.
